# New Jersey State Dental Society

**Published:** 1889-11

**Authors:** 


### NEW JERSEY STATE DENTAL SOCIETY.—(*Continued.*)

### DISCUSSION ON DR. RUSSELL'S PAPER ON "COPPER AMALGAM."

*Dr. Watkins.*—In regard to the oxidation of copper amalgam, I saw a large filling the other day that was six months ago put in a molar tooth; the tooth was mostly built up with copper amalgam. The canals were filled, but for some reason the tooth ulcerated, and I had to extract it. In the extraction the crown was broken off; a small piece of enamel which came off was yellow; the lower part of the filling was as clear as any enamel filling could be, while the outer surface was as black as ink. I had supposed that the under sides of the filling would oxidize the most, and that the oxide of copper becoming embedded in the dentine would perhaps prevent the recurrence of decay; but the condition of things in that filling was exactly opposite to what we would expect on that theory.

With respect to copper amalgam setting quickly, Dr. Russell stated that he was accustomed to squeeze out the excess of mercury with pliers. I never had very good success in that way with any amalgam. I do not like to squeeze amalgam. The best success I have had has been in taking care not to overheat the amalgam. I think the mistake is often made of heating it too much and evaporating too much of the mercury; then they have to add mercury, and, getting too much, must squeeze the excess out; and the result is a bad filling. If the amalgam is heated to the right consistency, then ground vigorously and rapidly in a wedgewood mortar, it will work very nicely. If it should require more heating, heat it a little, not sufficient to bring out globules of mercury on the surface, and grind it again. I have never seen any copper amalgam so dry that I could not work it. Two weeks ago, in Boston, I gave a clinic, and the Boston men were much interested, and asked a great many questions. They were surprised to see me work so hard in grinding the amalgam. They asked me to show them how to mix copper amalgam, and I did so. Dr. Fillebrown said he believed he could now use copper amalgam successfully. It is hard to get men into the way of grinding amalgam with vigor and a vengeance; and it must be so ground in order to get the best results.

I do not think there is any shrinkage whatever in copper amalgam, nor any swelling, and no chipping unless you leave too much of a feather edge.

*Dr. E. Parmly Brown.*—I cannot keep quiet when these questions are up. Looking back over the history of amalgam forty or fifty years, I remember how our fathers, and the fathers of American dentistry,—Dr. Leslie and Dr. Parmly and others,—argued against the use of amalgam because it was poisonous. It is poisonous, but I am using it right along. It kills bacteria. Bacteria do not develop worth a cent around a black amalgam filling. But if you want to make sure of it, use a weak solution of bichloride of mercury.

*Dr. Foote.*—I have had but very little experience in the use of copper amalgam, but I want to get the experience of those who have used it, in contradistinction to what is known as copper alloy. I have had some experience with copper alloy, ground and mixed with mercury. The instructions are to mix it soft and wafer it up. Quite a number of fillings put in in that way have come back in a softened condition months afterwards; so soft that they have been scooped out by the brush in cleaning the teeth, although I left them quite hard when wafering them up.

*Dr. Edmunds.*—I suppose copper alloy and copper amalgam are so separate and distinct that it is hard to draw a comparison between them. I do not understand how the gentleman's alloy fillings could be scooped out. They must have contained a large amount of copper, which would make a very soft filling.

*Dr. E. Parmly Brown.*—It strikes me now that we can look ahead in this copper amalgam business. Who can say that copper amalgam, with its antiseptic qualities in the mouth, is not going to prevent a thousand contagious diseases that may approach us, by killing the bacteria? Who can say that a thousand lives may not be saved by the slight antiseptic qualities carried in the copper amalgam fillings that we put in?

*Dr. Sanger.*—I was struck with one thing in the paper,—that is, that, contrary to general usage, the essayist did not claim copper amalgam to be good for everything. He admitted it was good in certain cases only, as I have found it in my experience. I simply rise to corroborate what he has said, and to tell my failures, the cause of which he has explained. In the use of copper amalgam I meet, every once in a while, with a very disastrous failure. At first I attributed them to my ignorance of the proper way to manipulate it. I noticed that the failures were bright fillings which had pleased me greatly when I looked at them a week or two after they were put in, their color was so good; but in a little while I found that my beautiful fillings were disappearing, and the edges showed signs of leakage. I believe Dr. Russell has explained clearly the cause of it. I found it invariably the case that where there was a strong acid reaction in the mouth my fillings failed; and when that condition did not obtain, my copper amalgam fillings were successful, and successful where other amalgam fillings failed.

*Dr. Gilson.*—Mr. President, I have noticed, in the use of copper amalgam, that the temperature of the weather when you mix it makes a difference in its working qualities. If you mix it on a cold day and in a cold mortar, you do not get the same result that is found when the mortar is slightly warmed. The temperature of the mortar should be about the same as that of the amalgam when we take it from the spoon. When the mortar is warmed you need not heat the amalgam quite as warm as would otherwise be necessary. Working it in the hand also tends to keep it warm, by friction and the heat of the hand.

*Dr. William H. Trueman.*—I have had some years of experience with copper amalgam, and have used a good deal of it. I admired one word that Dr. Russell used in his essay,—that is, the word discretion. I think copper amalgam should be used with a *great deal* of discretion. It is certainly a very valuable addition to our tooth-saving materials, but unless it is used with caution it probably will do about as much harm as good.

In order to get a fine precipitate of copper I found it necessary to have a solution of sulphate of copper, a very dilute solution. After obtaining the precipitate I wash it a number of times in a weak solution of boiling hydrochloric acid, till the acid no longer shows a chemical trace of iron. It is astonishing how long that washing has to be continued. Then it is put in a mortar and mercury is added. It is usually necessary to add a little hydrochloric acid, or sulphuric acid. Then the real work begins. It is remarkable how much black something can be washed out of a batch of copper amalgam before it can be called clean. It has usually taken me about two evenings, of about five or six hours each, to wash a batch of copper amalgam, using hot water, and changing the water an indefinite number of times. It is an exceedingly tedious process, but when the amalgam is clean it pays for all the labor expended upon it. Not only is it made very clean by this washing, but the constant trituration seems to improve its qualities very much. I find it better to keep quite an excess of mercury during this process. After it is completed the excess of mercury is pressed out through a chamois skin; then it is ground up again, and the surplus mercury again pressed out and removed. By this means you get the copper almost back in its original condition as a precipitate. I think it is best to leave the excess of mercury in the amalgam till you are about to use it.

When you first use copper amalgam you fall in love with it. It has certain qualities that are very pleasant. In the first place, it does not shrink. It can be used very soft, and then it becomes very hard. I think the reason it will remain in cavities and preserve teeth where nothing else will is because of its hardness, and this hardness does not depend very much upon the quantity of mercury in it. If you use a common amalgam, or amalgam alloy with an excess of mercury, it never becomes hard, and you have retaining grooves filled with an amalgam that has less hardness than the body of the filling. But as the hardness of copper amalgam does not depend so much upon the quantity of mercury it contains, the fillings made of it are retained in place more perfectly than alloy amalgam fillings would be.

The time was when I said I had not seen a case where copper amalgam had discolored the teeth. I cannot say that now. It is remarkable how long a tooth will retain its color, while the amalgam on the exposed surface becomes black; but after a time there is a staining of the tooth. It may be that the color in the amalgam shows through, but I am inclined to think that the tooth will in the end be permanently discolored.

It is a very nice thing theoretically to fill one portion of a cavity with copper amalgam and then upon that place gold. I have done that in some cases, and have found the teeth turning a very dirty green, which was very objectionable. You will use copper amalgam perhaps three or four years before you discover these things.

In regard to using an alloy with copper amalgam, I have never found alloy and amalgam to harden. In every case it has remained soft and granular.

There is another point in connection with its use with other metals. The nickel screws which are used to set crowns are sometimes entirely destroyed by contact with copper amalgam. After the lapse of twelve months or two years I have found the spaces that the nickel screws should have occupied entirely vacant.

I think Dr. Russell has done us a great service in advocating the use of copper amalgam with discretion. I use it, and expect to continue to use it. I think it will save teeth when nothing else will, but it should be used with a great deal of discretion.

Subject passed.

DISCUSSION ON DR. EDMUNDS'S PAPER ON "REMOVABLE BRIDGE-WORK."

*Dr. E. Parmly Brown.*—Mr. President, ladies, and gentlemen: When I promised to reply to Dr. Edmunds's paper I had no data to guide me in my remarks, until I received the *American Journal of Dental Science*, which had a paper by Dr. Edmunds on crown and bridge-work, copied from the *Student's Record.* While reading that paper I noted down a few points, thinking that the doctor would probably put some of the most salient in this paper. I afterwards received from him what he called a synopsis of his paper, but from it I could get very little to go by. I find, to my chagrin and discomfiture, that he has not put any of the points of that paper into his paper of to-day. His paper is all right; I cannot take any exceptions to it. I have not tried rubber nor celluloid for bridges. Rubber is good for certain purposes. A removaable bridge, one that may be removed, but does not move when it is not wanted to be removed, would be a grand thing. The majority of the bridges made to-day are removable long before you wish them to be. As they told me over in England, most of them were "'anging haround hon one 'inge" in a few weeks. But that is not saying that bridge-work is no good in itself. If one of your fillings should happen to come out, you would not say, "I will never insert another filling; fillings are no good." Here is a gentleman at the door (Calvin) who is in danger from implantation. I told him that although freedom took him off the plantation as a slave, yet he has to go back on a plantation because he has one in his mouth, and the first thing he knows he will be a slave again. The question will come up, Who is he? When Webster killed Parkman in Boston, they discovered the murdered man by his teeth found in the furnace. Now, suppose the person from whose mouth that tooth of Calvin's was taken should be murdered, and her teeth should be discovered in his mouth; why the jury would find that that young lady had been murdered by our friend from Central Africa. If we are going on in this way to wear somebody else's teeth, I should like to know who we shall be in the course of time. Dr. Edmunds said that bridge-work made of rubber and celluloid could not be detected on the most careful examination.

*Dr. Edmunds.*—No; I said observation.

*Dr. Brown.*—Observation; at a distance——

*Dr. Edmunds.*—While speaking to the person.

*Dr. Brown.*—I have been mussing with teeth for nearly forty years, since I was six years old, and I have never yet seen an artificial tooth that was backed up with a material like gold, rubber, or any material of that kind, that was not translucent or transparent, and that had at a long range a natural appearance, let alone close observation such as you get in conversation.

*Dr. Pinney.*—If Dr. Edmunds would show us some samples of his work, which he speaks of, we should be able better to understand his ideas and to discuss his paper more intelligently.

*Dr. Edmunds.*—I have here only the casts on which some of the work was done; I have not a sample of the work.

*Dr. Watkins.*—I am informed that the teeth which were used to retain the plate in the case described were loose, from the effects of salivary calculus, before the doctor began to treat them; he treated them, and new granulations were thrown out and the teeth have become firm, and on those two roots he placed the bridge-work. He had more courage than I should have had under the circumstances.

*Dr. Edmunds.*—They were nearly firm when I capped them. They are now as firm as they were when the bridge was inserted. The piece was put in a year ago last June.

*Dr. Brown.*—That is a point in the paper which I want to speak of,—inserting bridges upon teeth that are loose. A good way to keep such teeth loose and prevent the parts from healing up would be to allow them to stand without support. The way to give nature a chance to heal them up and make them firm is to put them as soon as possible in the condition they were in when God implanted them there; to allow them to be supported in the dental arch; and the quicker you can get those roots bridged or crowned, no matter if there are running abscesses around them, the quicker they will heal. Three days ago a lady came to me with an abscess; she had had an artificial crown inserted to hold a bridge, and the pin of the crown had been allowed to run up about half the length of the canal. Nature gave us a long canal to support crowns, and when the dentist in fastening a bridge or crown cuts off the pin and puts it in but half the length of the canal, he fixes up a very nice arrangement for splitting the root. Put the pin in as far as the canal will permit, and you get strength, and the root will not split half so easy as it will if the pin goes up but half the distance. I lanced that abscess and cauterized the opening. I said, that is a safety-valve. Then I inserted a crown, running the pin entirely to the apex of the root. The next day there was no external sign of inflammation at the root.

With regard to sustaining a loose tooth with a bridge: if you have other healthy piers to support it, you are saving that individual root from being attacked alone by its enemy. The things which move it prevent nature from healing it. In union there is strength. We ligate loose teeth for the purpose of giving nature a chance to heal them. Remove the irritating cause, keep them firmly in position, and they will heal nicely.

I have in mind two cases where I inserted porcelain dentures. In one case there was a single canine root. It was very sore. I knew it would get well quicker if we could hold it in position than if we were to apply powerful medicines. The work was done at once, and the tooth got well immediately. I saw the case a few days ago, and the root is perfectly healthy.

*Dr. Stockton.*—Mr. President, I am sorry that Dr. Brown has not spoken more particularly upon the principal point of the paper, which I take to be the advantage of a removable bridge over a fixed one. That is what I wanted to hear Dr. Brown discuss. So far as I am personally concerned, I may say that I have seen a great many cases of fixed bridges, and my impression is that they are a nuisance to the patients and to those that come in contact with them, and that they sooner or later cause loss of the teeth to which they are attached. If bridges can be made removable, it seems to me that that would be a great deal better way of putting them on. The only question in my mind is whether the constant taking out of the bridge for cleansing purposes would not cause loosening of the capped teeth. I have not seen removable bridges in use long enough to determine that question. But I am satisfied that bridges that are so fastened that they cannot be removed from the mouth by the patient are not a good thing; and if we must wear an artificial substitute of some kind I would much prefer a good gold plate to a fixed bridge that could not be taken out and cleansed. Our mouths are dirty enough anyhow, the best of us; the mouths that come to us are oftentimes anything but sweet and pleasant; and if we place in them an artificial denture that cannot be removed and cleansed, it will surely prove to be a nuisance. It is on this point especially that I wanted to know Dr. Brown's opinion.

*Dr. Edmunds.*—Dr. Stockton has confined himself closely to the point that I wished to make in my paper. After wearing a fixed bridge in my mouth for five years, I found that, although there were only a few teeth attached, it was almost impossible to keep the piece as clean as I should like. It occurred to me to make a removable bridge; and after several experiments I made one which was held perfectly in place, without rubbing the teeth that it was attached to.

When I spoke of making the capped tooth conical from the margin of the gum, I did not mean that I made it completely so; it is rather flattened on the anterior and posterior surfaces, which prevents the bridge from being twisted around in the mouth. There is no additional strain upon the teeth that support the bridge, because the bridge rests upon the gum, and the force of the other jaw comes upon the gum.

*Dr. Brown.*—I did say something about removable bridges. I said I did not know what Dr. Edmunds's bridge was. I will now say that if a removable bridge is adopted by the dental profession in preference to an immovable one,—if we are not equal to the occasion and want to bridge an intervening space,—they are the best thing. By having a removable bridge we save ourselves work; we can make a lady's teeth so she can take them out and give them to the bootblack to be cleansed, and then stick them in again. Removable bridges are good; that is not saying that immovable bridges are not better. When we began to adopt bridgework I looked into it, and I saw it was difficult; I knew that the common herd would never go ahead and lead, but would be content to walk the beaten path. It takes a man to discover America, or to go through the wilds of Central Africa; it takes a man that will not be rebuffed by the first savage tribe or tiger he sees. It takes a man of nerve to make any advance in dental practice that is worth anything; it takes a man that will not be discouraged by one or two failures, but will look farther and discover whether those failures can be avoided, and consider whether the percentage of failures is in accordance with the percentage of successes that will bless mankind.

*Dr. Pinney.*—I have been unfortunate enough to lose a tooth or two, making it necessary to bridge over that space. At one time I had what I thought was the very finest thing out, a removable bridge, but it soon started the tooth to which it was attached, and wiggled it out. It was the work of one of the very best workmen perhaps in the country. After that I had a permanent bridge made, and it is one of the most beautiful things I have ever seen. It is a great deal more cleanly than the other was. I could not keep the removable bridge clean, and it would shift about and bother me all the time. I was happy when the tooth came out and I could throw the thing away. The permanent bridge is a great satisfaction to me. I do not think I have ever seen a removable bridge that was worth anything. You cannot keep it tight. It wiggles a great deal, and finally the tooth comes out, or something is wrong about it. The permanent bridge stays in place without any trouble.

*Dr. Truax.*—This subject of bridge-work is like the old one of filling teeth. Some will use nothing but gold, and others tell us nothing but amalgam should be used; some prefer gutta-percha, and others like a combination. After all, the question reduces itself to the filling of teeth with judgment in each individual case. The removable bridge has its advantages. The advantage of the removable bridge is that it can be taken out and cleansed. I think we should learn to discriminate between them, and to use the right one in the right place, making our work an individual operation in each case, which will insure higher results and the greatest good to patients.

*Dr. Edmunds.*—In reply to Dr. Pinney, I shall be happy, at some future time, at some meeting of the District Society of New Jersey, or at your annual meeting, if you will send me a notice, to produce at the clinic or society meeting a patient for whom I have inserted one of these bridges, and I think an examination of the work will convince every one that a removable bridge, of any dimensions, can be kept perfectly clean, and will not wiggle about in the mouth as Dr. Pinney describes. It can be kept cleaner and better than immovable bridge-work.

*Dr. Stockton.*—This is a very interesting question, the comparative merits of removable and immovable bridges. We have with us Dr. Waters, who presented to the profession a removable bridge; he has had very large experience undoubtedly, and I think you would all be very glad to hear him on this question.

*Dr. Waters.*—Mr. President, my experience has been that removable bridge-work is in my hands much preferable to fixed bridge-work; it has been more satisfactory to my patients and to myself. I have constructed both kinds in the mouths of patients whom I knew to be cleanly, and my experience has led me to make removable bridges wherever it is practicable. We cannot make a practical piece of removable bridge-work in all cases, nor can we make a thoroughly practical piece of fixed bridge-work in all cases. We must exercise our judgment as to which is adapted to the particular case in hand. I have had several pieces of removable bridge-work in the mouth for some years. Dr. Brown has seen one piece after it was worn a considerable time. I received a letter in June from a young man who had been wearing for two years a removable bridge which I made for him, saying he had had the misfortune to have it broken. While he was cleaning the piece a young man, seeing some disturbance on the street, ran by and knocked it out of his hand and put his foot upon it, and disfigured it very much. He was much distressed, as he was anxious to go to the Georgia State dental meeting and wanted to exhibit that piece of work. He sent me the wreck, together with an expression, model, and articulation. In about a week from the time he sent it to me he again had the piece in his mouth, and he wrote me that it was as satisfactory as the former piece. I made it without seeing the patient. A strong point in favor of removable bridge-work is that a patient at a distance can send an impression and model to you and have a new piece made without having to visit you personally; and in case of any accident to the teeth by which it is supported the piece can be removed and the teeth treated. With fixed bridge-work, in case of any accident, the entire piece must be removed, which requires considerable time and work. One of the patients I spoke of as being very cleanly had the misfortune to break a piece of fixed bridge-work in trying to remove it. I afterwards put in his mouth a piece of removable bridge-work, since which I have had no trouble with it, nor has he.

One feature of Dr. Brown's method is much in its favor; that is the porcelain. I have found that where gold is exposed in the mouth it is very difficult to keep it clean, but with porcelain there is not much difficulty. I have not seen any of his cases.

I do not know that I can say anything more, except that in my hands removable bridge-work, constructed with judgment, is preferable to fixed bridge-work. Removable bridge-work is certainly an improvement over the old clasp method.

In the case that I spoke of, the young man wears a molar, cuspid, and bicuspid, supported by a molar and bicuspid, and those teeth are as firm now as they were when they were capped. He wrote me that after the bridge had been off for about a week the supporting teeth got a little tender, but after the piece was put back in place they felt all right. Whether taking a bridge out two or three times a day to cleanse it would tend to loosen a supporting tooth in course of time remains to be seen. I have one case that has been worn for three years, and the teeth are in perfect condition and the whole work very satisfactory.

*Dr. Pinney.*—I would like to ask Dr. Waters and Dr. Edmunds how long it will take the saliva or the acids of the mouth to become putrid around those caps. It takes but a very short time; and I have yet to remove a bridge or cap, or anything of that kind that had been worn a few hours, that was not very offensive. You cannot make the caps so tight as to exclude the fluids of the mouth, and in an hour or two they are rancid. I think the case of Dr. Waters, which was sent a long distance to be repaired, is not a very great triumph over fixed bridge-work. If he had not had his teeth out of his mouth, no person could have put his foot on them and they would not have been broken.

*Dr. Edmunds.*—The caps can be made absolutely air-tight, and the saliva will not accumulate under the cap over which it is telescoped, if made in the way I have suggested.

*Dr. Rhein.*—I suppose the best of us are too frequently wont to go ahead and do operations too quickly, without judging the best method to be followed in each case. And we are, of course, somewhat excusable in so doing, because our time is valuable, and because a great many of our patients are hardly willing to pay for time taken in reflection; but that is where most of the errors are made in doing anything that is not a simple operation. We do not take into consideration whether this thing would be better than that, or some other operation better than either of them. If we were to do that, we should generally reach a conclusion that would not afterwards be found to be a failure.

The point I wanted to raise in this matter was the question of the possibility of keeping fixed bridges in a cleanly condition. I have seen a number of fixed bridges that were in a very filthy condition; yet I have seen a number of them in as cleanly a condition as most patients keep their natural teeth; and the impression I have gained has been that in every case where the operator has endeavored to make a cleansing space under the fixed bridge he has made a place for the accumulation of filth and *débris*, sooner or later. Even where a decided cleansing space has been left, the soft tissues will, in course of time, close up that space just sufficiently to make it exactly the opposite of what it was intended to be. I believe the only successful cases of fixed bridge-work that I have seen have been those in which the bridge impinged so tightly upon the soft tissues as literally to embed itself in them. That is the method that I have always used.

Dr. Waters has expressed the opinion that porcelain is much more cleanly than gold, and I think he is quite correct in that. He, however, thought the gold fastening of porcelain bridges to the natural teeth was an objection. It is no more a bar against porcelain bridge-work, if it is perfectly adapted to the tissues and embedded in the gum, than an artificial gold filling in a natural tooth. If a filling is made in such a way as simply to contour the space between the natural teeth and the bridge, leaving a space in some cases, perhaps that would be self-cleansing; in other cases there would be no space at all.

*Dr. E. Parmly Brown.*—It is a very healthy sign, gentlemen, to see you sit here so long listening to that awful subject of bridge-work. Only one gentleman has said nothing whatever against bridge-work, and that is Dr. Stockton, and I will venture to say that he has never put in one. Every man who has adopted bridge-work, and who has done it successfully, backs it up, so far as I know..

Dr. Waters presented, I was going to say, some splendid arguments in favor of removable bridge-work. It is a splendid piece of work; better than any plate, or anything else except a piece of good porcelain bridge-work, permanently fixed, and resting so firmly on the gum as to smother bacteria to death. That is what you really want, and what will do you good.

*Dr. Stockton.*—Mr. President, the remark of Dr. Brown, that the only gentleman who has not raised his voice against bridge-work was Dr. Stockton, and that he probably had never used it, is in accord with many of his statements,—they are to be taken with a very large dose of salt. I have put in a great many pieces of bridge-work, and I did not raise my voice against bridge-work, nor do I. The only question I raised was, Which is the better of the two methods? Bridge-work is used, and will be used for all time to come, and what we want to know is which is the better plan of putting it in. It has been said that by keeping loose teeth in a rigid position they were likely to become firm and good, and it is true that tying a loose tooth to a firm one is the best way to get it firm. But the question arises whether the frequent removal of these bridges, which are made so tight-fitting that the saliva cannot get under the fastenings, will tend to loosen the teeth to which they are anchored. That is the point on which I should like to have Dr. Edmunds's opinion.

*Dr. Edmunds.*—The case that I spoke of in my paper seems to be a sufficient refutation of the notion that the removal of the bridges for cleansing purposes will loosen the supporting teeth. In that case the teeth were loose when I put the bridge in; they were in bad condition; and to-day, after the bridge-piece has been worn about three months, they are more firm than they were the day I inserted it.

*Dr. Carroll.*—I did not hear the paper. I rise merely to reinforce some remarks made by Drs. Rhein and Brown, with regard to the relative value of fixed and removable bridge-work. While I am a practitioner of the old school, and somewhat fixed in methods of work, I agree with Dr. Rhein as to thorough eclecticism in the use of bridge-work. The man who undertakes to pursue one form of bridge-work in all cases will have failures. I have adopted a special form of fixed bridge, made by capping the abutment roots or teeth, and then telescoping over them, resting the bridge not only upon the abutments, but firmly upon the gum as well. Theoretically that is not a good form of bridge-work. Practically it is *the* form of bridge-work, to my mind, to-day, and the only form that is going to stand in all cases. Theoretically it is not a good form, for it is a saddle or plate resting upon the gums, and therefore might become foul. I adopted that form very reluctantly. During the past eighteen months I have put some thirty or forty different pieces of this kind in my patients' mouths, watching them very closely. The last case I observed was yesterday in my office, one of the most cleanly patients I have ever had in my practice. He had nothing back of the cuspid, but had the teeth in front of the cuspid. I saddled the lateral and the cuspid on one side, and the cuspid and bicuspid on the other side,—the most unfavorable form for a fixed bridge. They were aluminium bridges. When cemented in place upon the teeth they rested firmly upon the gums, and having a good occlusion below, he had excellent use of it as a mill with which to grind. I have seen the patient a number of times since it was inserted, and he says there is no uncleanliness whatever. In my own mouth I have a fixed bridge of the kind indicated, and I have a constant sense that it is not a cleanly piece of work. It was made by one of the best workers in America. On the other side I have a removable bridge, which I can keep pretty clean by removing and cleaning it. The result of my observation in over fifty cases of bridge-work is that the fixed form is the best when resting not upon the teeth alone, but upon the alveolus as well.

On motion of Dr. Watkins, the subject was passed.

